# Targeting glutaminase1 and synergizing with clinical drugs achieved more promising antitumor activity on multiple myeloma

**DOI:** 10.18632/oncotarget.27243

**Published:** 2019-10-15

**Authors:** Qiang Qiu, Mengyuan Li, Linyu Yang, Minghai Tang, Li Zheng, Fang Wang, Huandi Qiu, Cailing Liang, Ning Li, Dongni Yi, Yuyao Yi, Cong Pan, Shengyong Yang, Lijuan Chen, Yiguo Hu

**Affiliations:** ^1^ State Key Laboratory of Biotherapy and Cancer Center, West China Hospital, Sichuan University and Collaborative Innovation Center, Chengdu, Sichuan, China; ^2^ Department of Hematology, West China Hospital, Sichuan University, Chengdu, Sichuan, China; ^3^ Department of Thyroid Surgery, West China Hospital, Sichuan University, Chengdu, Sichuan, China; ^4^ Guizhou Normal College, Guiyang, China

**Keywords:** glutaminase1, multiple myeloma, BPTES, cMYC/KRAS12V-transduced plasmacytoma, mouse model

## Abstract

Multiple myeloma (MM) pathogenesis remains incompletely understood and biomarkers predicting treatment response still remain lacking. Here we describe the rational mechanisms of combining targeting glautaminase1 (GLS1) with other chemo-reagents for MM treatment. Gls1 is highly expressed cMYC/KRAS12V-drived plasmacytoma (PCT) cells. Down-regulation of Gls1 with miRNAi in cMYC/KRAS12V-expressing BaF3 cells prevented them from growing independence of interleukin 3 (IL3). By using our cMYC/KRAS12V-transduced adoptive plasmacytoma mouse model, we found that Gls1 is involved in PCT pathogenesis. Down-regulation of Gls1 significantly prolonged the survival of PCT recipients. Knockdown of Gls1 increased the expression of Cdkn1a and Cdkn1b and decreased the expression of some critical oncogenes for cancer cell survival, such as c-Myc, Cdk4, and NfκB, as well as some genes which are essential for MM cell survival, such as Irf4, Prdm1, Csnk1α1, and Rassf5. Combination of Gls1 inhibition with LBH589, Bortezomib, or Lenalidomide significantly impaired tumor growth in a MM xenograft mouse model. Our data strongly suggest that Gls1 plays an important role for MM pathogenesis and that combination of GLS1 inhibitor with other MM therapy agents could benefit to MM patients.

## INTRODUCTION

MM is the second most common hematological malignancy affecting millions of people worldwide [1]. High dose chemotherapies, as well as immunotherapies, have significantly improved the response rates and survival outcomes in MM patients [2, 3]. However, MM still remains incurable, indicating a strong need for continuing investigation for developing innovative therapeutics. Altered cancer cell metabolism as a common event in cancer progression has been long recognized. A hallmark of metabolic reprogramming is the increased utilization of glucose and elevated lactate production even in the presence of oxygen and is known as the Warburg effect [4]. Targeting the Warburg effect is becoming a useful strategy for preventing or stopping the development of cancer [5, 6]. Previous studies have demonstrated that many genes are associated with the Warburg effect, including those involved in glycolysis and glutamine metabolism [7, 8]. As the essential enzyme for glutamine metabolism, GLS1 is a potentially critical target for cancer therapy. GLS1 is an amidohydrolase enzyme that catalyzes hydrolysis of glutamine to glutamate and ammonia. The resulting glutamate is acted on by glutamate dehydrogenase (GDH) or transaminases to produce a-ketoglutarate (α-KG) for the citric acid cycle. Glutamine metabolism restriction with a specific inhibitor is effective in inhibiting tumor growth both *in vitro* and *in vivo* [9–11]. Generally, GLS1 is highly expressed in many types of cancers, including MM, and previous studies have shown that inhibition of glutamine metabolism impairs MM cell survival and overcomes drug resistance *in vitro* [12–14], but the detailed mechanism *in vivo* are still insufficient.

It is well known that several genes are critical for establishment and progression of MM including MYC, RAS, NFκB, IRF4, XBP1s, Csnk1α1, CCND1, and others [15–23]. MYC plays a critical role during MM pathogenesis. Ectopically expressing MYC alone in mature mouse B cells can produce malignancy [24]. Multiple rearrangements and activated of MYC also occurs in the transition of monoclonal gammopathy of undetermined significance (MGUS) to MM [19]. Mutations of RAS members are the most common oncogene mutations found in MM [17, 18]. NFκB pathway is also critical for MM survival and proliferation. Several signaling pathways, such as APRIL and BAFF ligands and their receptors, which are important for MM pathogenesis, directly activate NFκB pathway [25, 26]. Csnk1α1, participates in Wnt signaling, which is essential for malignant plasma cell survival in cMYC/KRAS12V-transduced PCT mouse model [23].

GLS1 was upregulated in primary myeloma cells isolated from MM patients and played an important role in MM cell growth and survival [14]. However, the detailed mechanisms are still poorly understood and several critical issues remain unknown, for example, whether targeting GLS1 could prevent MM initiating and completely eliminate cancer cells *in vivo*.

Here, we demonstrated that downregulation of Gls1 with miRNAis could significantly prolong mice survival but not prevent cMYC/KRAS12V-transduced PCT initiation by using our mouse model. Combination of Gls1 inhibitor with current MM therapy drugs can achieve synergic cytotoxicity effects on MM cells both *in vitro* and *in vivo*. It provides the rationale for future clinical trials of targeting GLS1 to improve the outcome of multiple myeloma patients.

## RESULTS

### Gls1 was required for cMYC/KRAS12V transforming BaF3 cells independent of IL3.

**Figure 1:**
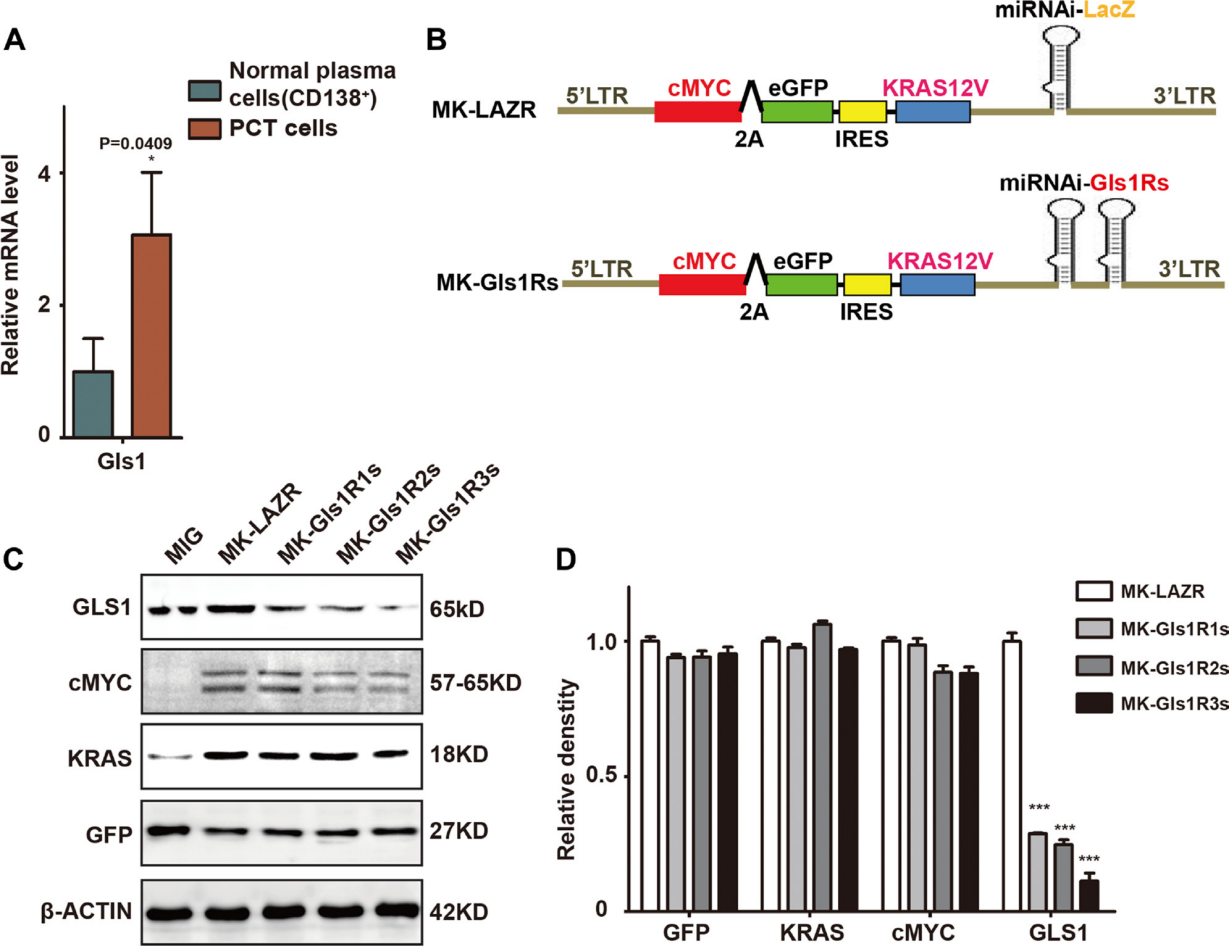
GLS1 was required for cMYC/kRAS12V transforming BaF3 cells independent of IL3. (**A**) Gls1 expression is increased in cMYC/KRAS12V-transduced PCT cells when compared to normal plasma cells (CD138^+^) with RT-PCR, *P <* 0.05^*^(*n* = 3, *t* test). Data presented are from three independent experiments and presented as mean ± SE. (**B**) Schematic diagram of MSCV-based retroviral vectors: cMYC/KRAS12V with miRNAs targeting lacZ and Gls1, GIs1Rs means miRNA targeting GIs1 sequence repeats. (**C**) MIG, MK-LAZR, and MK-Gls1Rs vectors as indicated were transfected into 293T cells and total protein was analyzed for GLS1, cMYC-2A-eGFP, KRAS12V, and GFP expression. β-actin served as loading controls. (**D**) Relative density of GFP, KRAS, cMYC and GLS1 protein in (**C**), *P <* 0.0001^***^(*n =* 3, *t* test).

Previous studies have demonstrated that inhibition of glutamine metabolism impaired MM cell growth and survival *in vitro* [14]. Compared to normal plasma cells isolated from syngeneic mouse spleen, Gls1 expression was elevated in cMYC/KRAS12V-transduced PCT cells (Figure 1A). To investigate whether Gls1 was involved in PCT pathogenesis, we employed the cMYC/KRAS12V-transduced adopted PCT mouse model. Three vectors containing the miRNAis targeting both human and mouse Gls1 mRNA were constructed (Figure 1B). Gls1 was significantly knocked down by all miRNAis, as compared to scrambled sequence in transfected 293T cells (Figure 1C–1D).

To examine whether all elements in these plasmids functioned as expected, protein of cMYC, KRAS, eGFP, and GLS1 were examined in transfected 293T cells. We found that cMYC, KRAS, and eGFP were equally expressed by these plasmids and Gls1 was significantly knocked down by miRNAis (Figure 1C and 1D). These results suggested that all elements in these constructs were functionally the same as in our previous results [23].

**Figure 2:**
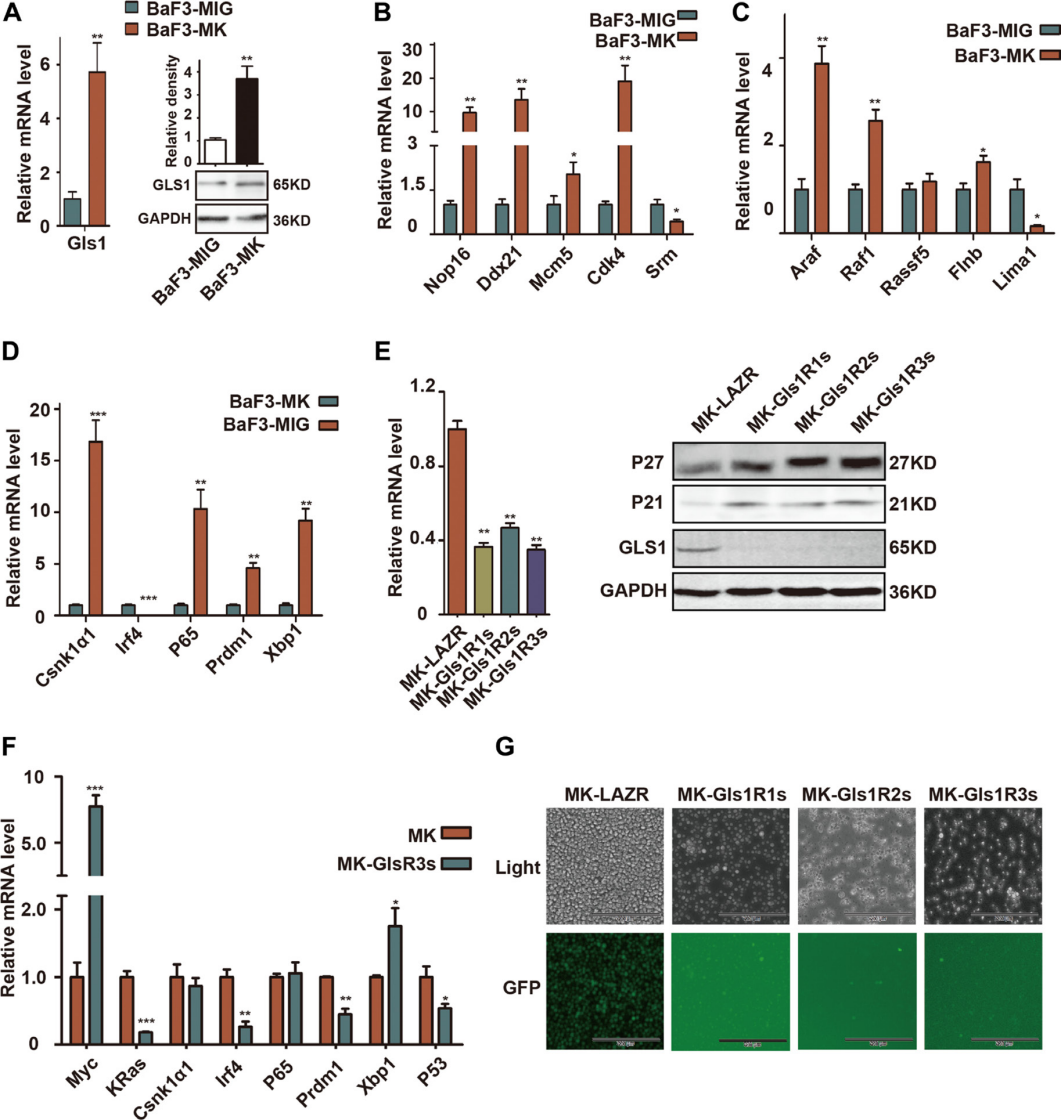
GLS1 was required for cMYC/kRAS12V transforming BaF3 cells independent of IL3. (**A**) RT-PCR and immunoblotting analysis shows expression of Gls1 in cMYC/KRAS12V-expressing BaF3 cells. (**B**–**D**) The expression of cMYC targets, RAS targets, and biomarker genes of MM in cMYC/KRAS12V-expressing BaF3 cells (**E**) Gls1 mRNA (left), GLS1, P21 and P27 protein levels (right) in MK-LAZR-, MK-Gls1R1s-, MK-Gls1R2s-, and MK-Gls1R3s-transduced BaF3 cells. (**F**) The expression of indicated genes in MK-LAZR-, and MK-Gls1R3s-transduced BaF3 cells. Data presented are from three independent experiments and presented as mean ± SE. P < 0.05^*^, *P <* 0.01^**^ and *P <* 0.001^***^ considered significant. (**G**) Down-regulation of Gls1 in BaF3 cells, cMYC/KRAS12V failed to drive transfected cells to grow independently of IL3. Representative cells from three independent experiments are shown.

Consistent with *in vivo* results (Figure 1A), both of Gls1 mRNA and protein were significantly elevated in cMYC/KRAS12V-transformed BaF3 cells (Figure 2A). To characterize the transformed cells, we selected 15 biomarker genes including cMYC or KRAS targets, MM biomarkers, or genes critical for MM cell survival and examined their expression with RT-PCR. Among selected 5 cMYC target genes, the expression of Nop16, Ddx21, Mcm5, and Cdk4 was increased and only Srm was decreased (Figure 2B). Similar results were found within KRAS target genes; the expression of Araf, Raf1, Rassf15, and Flnb was elevated and Lima1 was decreased (Figure 2C). Among selected MM biomarker genes, Csnk1α1, P65, Prdm1, and Xbp1 expression was significantly increased, and unexpectedly, Irf4 was downregulated in cMYC/KRAS12V-transformed BaF3 cells (Figure 2D).

To examine the biological sequelae resulting from Gls1 knockdown, MK-LAZR, MKGls1R1s, MKGls1R2s, and MKGls1R3s were retrovirally transfected into BaF3 cells. GFP^+^ cells were sorted and continually cultured with IL-3 for another 2-3 days. As expected, both mRNA and protein levels of Gls1 were significantly knocked down by these miRNAis in BaF3 cells (Figure 2E). Furthermore, the Cyclin-dependent kinase inhibitor P21 and P27 were obviously elevated (Figure 2E). The mRNA levels of some biomarker genes were measured at 24h post IL-3 withdrawn (Figure 2F). After IL-3 was withdrawn for another 5 days, only MK-LAZR drove BaF3 cell growth independent of IL3, but MKGls1Rs could not (Figure 2G). These data indicated that Gls1 was required for cMYC/KRAS12V to make BaF3 cell growth independent of IL3.

### Knockdown of Gls1 significantly delayed cMYC/KRAS12V-induced PCT pathogenesis in mice

**Figure 3:**
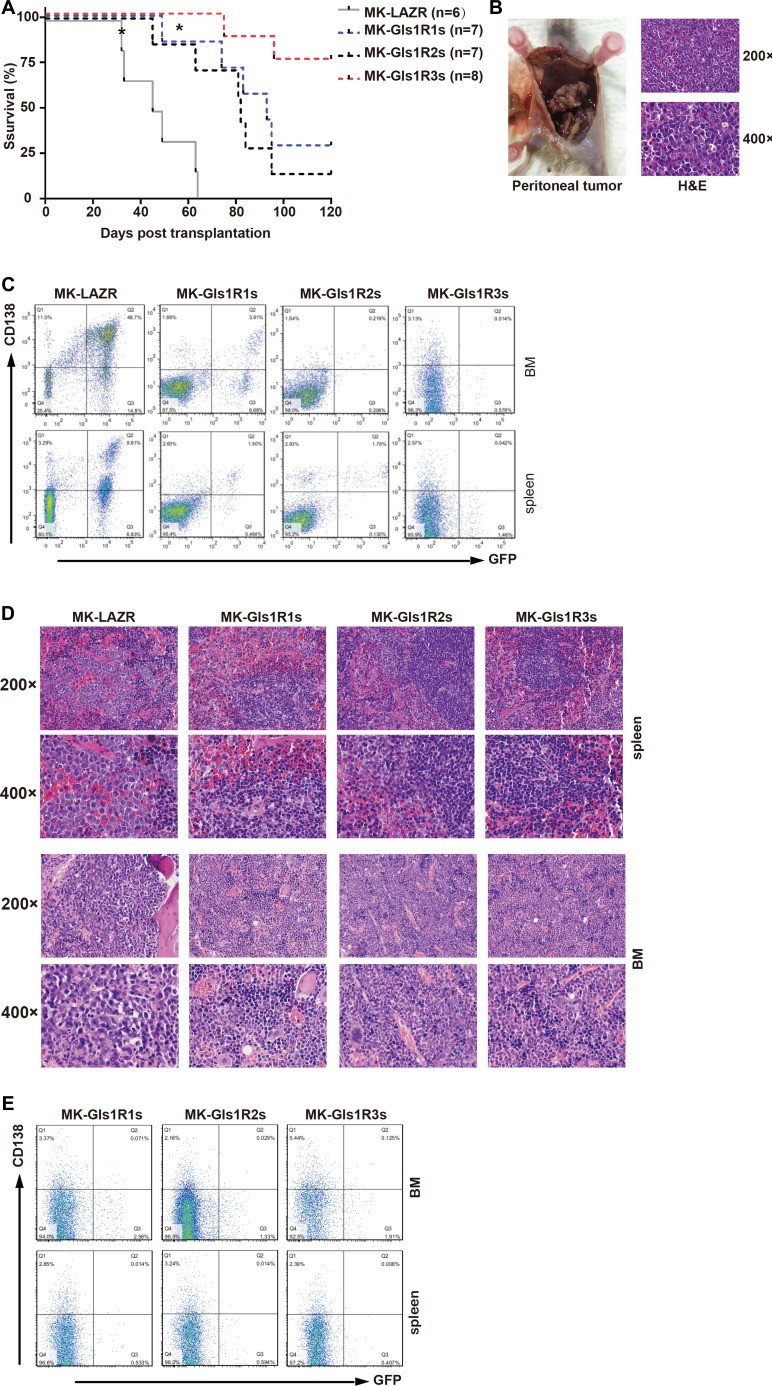
Down-regulation of Gls1 impeded cMYC/KRAS12V-transduced PCTs in Balb/c mice. (**A**) Kaplan-Meier-style survival curves for recipients received MK-LAZR- (grey line, *n =* 6), MK-Gls1R1s- (blue line, *n =* 7), MK-Gls1R2s- (black line, *n =* 7) and MK-Gls1R3s (red line, *n =* 8) transduced splenic IgM^+^ cells. Most diseased mice developed PCT excluding two recipients which developed B-ALL in MK-LAZR and MK-Gls1R1s groups, respectively (indicated with^*^). Two independent experiments were carried out with different viral stocks and all viral titers were equalized before *ex vivo* cell transfection. The difference in survival between MK-LAZR and MK-Gls1Rs is significant (*P* < 0.0001, Mantel-Cox test). (**B**) Tumors in the peritoneal cavity were pathologically observed in all mice groups Scale bars, 100 μm (top panel, magnification 200×) and 50 μm (bottom panel, magnification 400×). (**C**) FASC analysis is to track tumor cells in femur and tibia BM, and SPL. Numbers represent tumor cell percentages in respective gates. (**D**) Photomicrographs of BM and SPL sections (*n =* 3) from all group mice were stained with H&E. Scale bars, 100 μm (top panel, magnification 200×) and 50 μm (bottom panel, magnification 400×). (**E**) All the left recipients were sacrificed at day 120, BM and SPL cells were isolated and analyzed with FACS for GFP and CD138.

To determine whether Gls1 was involved in MM pathogenesis, cMYC/KRAS12V-transduced adoptive plasmacytoma model was employed. Most recipients (5/6) receiving MK-LAZR-transduced cells died of PCTs within 10 weeks post cell transplantation (Figure 3A). Recipients developed PCT identified by peritoneal tumors, ascites, and enlarged spleens with pathological characteristics and FACS findings (Figure 3B, 3C). In the control group, one recipient died of B-ALL, in which cells were typified by GFP^+^B220^+^ (data not shown). Although the median survival of Gls1 knockdown groups is over 6 weeks longer (Figure 3A), most recipients in MKGls1R1s and R2s groups died of PCT with the same pathological characteristics as LAZR recipients (Figure 3B, and data not shown). While most (6/8) recipients still remained healthy at day 120 post transplantation in MKGls1R3s group (Figure 3A). To monitor disease progression, at day 42, three recipients from each group were sacrificed for histology and FACS analysis. Overall, there were fewer and lower percentages of PCT cells in the SPL and BM sections from Gls1 knockdown recipients (Figure 3C and 3D). All recipients who were left were sacrificed at day 120; BM and SPL cells were isolated and analyzed with FACS for GFP and Cd138. No tumor cells (GFP^+^CD138^+^) were detected in these recipients (Figure 3E).

**Figure 4:**
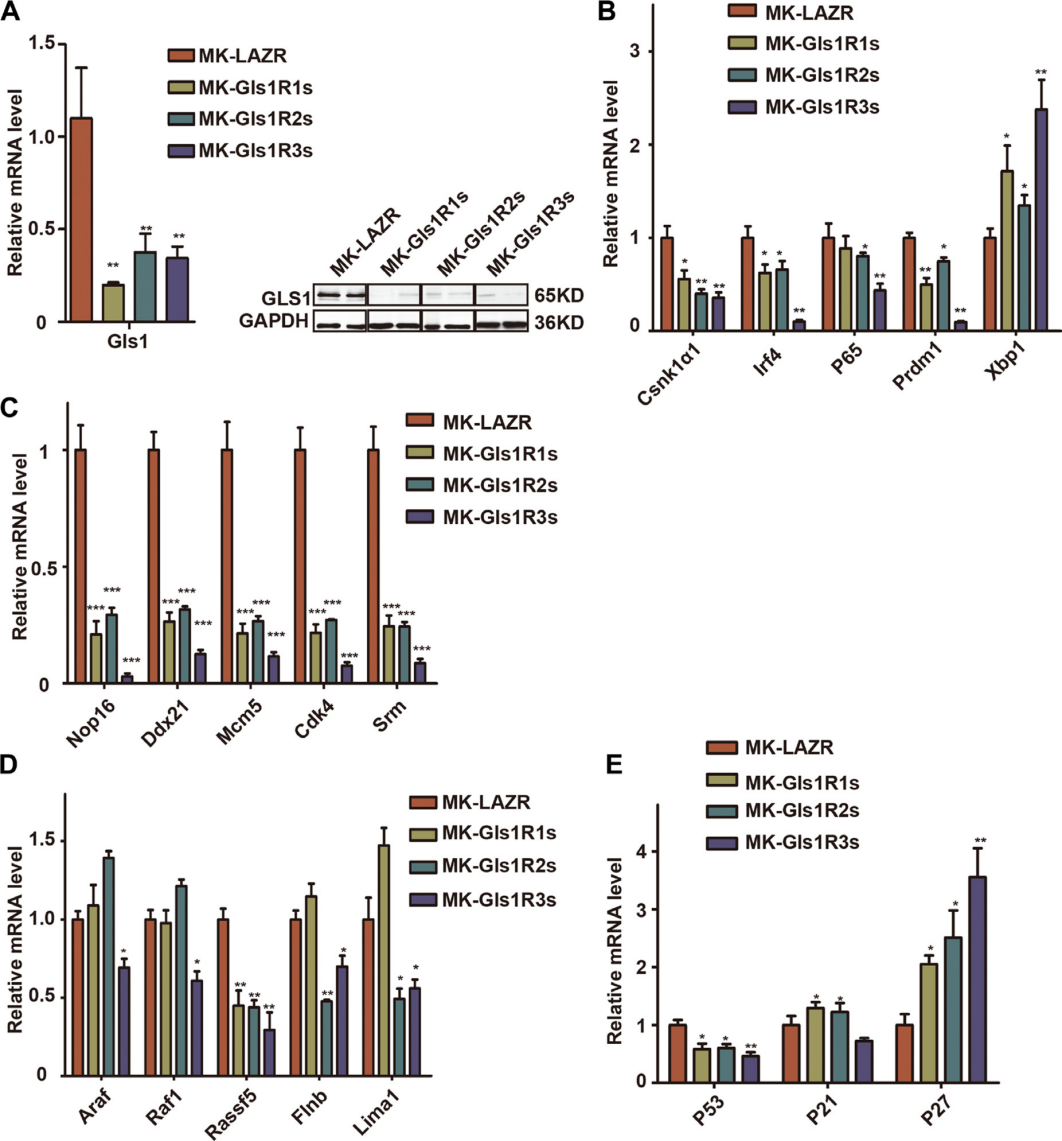
Down-regulation of Gls1 in PCT cells impaired signaling pathways important for MM survival. (**A**) Gls1 mRNA or protein level in MK-LAZR-, MK-Gls1R1s-, MK-Gls1R2s-, and MK-Gls1R3s-transduced PCT cells. (**B**–**E**) RT-PCR analysis shows expression of biomarker genes of MM, cMYC targets, RAS targets and key genes regulating cell cycle and apoptosis as indicated in MK-LAZR-, MK-Gls1R1s-, MK-Gls1R2s-, and MK-Gls1R3s-transduced PCT cells. Data presented are from three independent experiments and presented as mean ± SE. *P* < 0.05^*^, *P <* 0.01^**^ and *P <* 0.001^***^ considered significant.

Consistent with *in vitro* results, both mRNA and protein levels of Gls1 were significantly knocked down by these miRNAis in cMYC/KRAS12V-transduced PCT cells isolated from diseased recipients’ ascites (Figure 4A). We found that the expression level of *Csnk1α1*, *P65*, *Prdm1*, and *Irf4* was significantly down-regulated; whereas Xbp1 was increased (Figure 4B) in Gls1 down-regulated cells. All examined MYC targets, Nop16, Ddx21, Mcm5, Srm, and Cdk4 were decreased (Figure 4C). Among KRAS targets, only Rassf15 was down-regulated in all Gls1 knockdown cells. The expression of Lima1 and Flnb was decreased in Gls1R2s and Gls1R3s cells, but not Gls1R1s cells; Araf and Raf1 were only down-regulated in Gls1R3s cells (Figure 4D). We also examined several important genes for cell cycles or apoptosis, overall, Cdkn1a and Cdkn1b were up-regulated, and Tp53 was down-regulated in these Gls1 knockdown cells.

### Knockdown of Gls1 altered multiple signaling pathways in PCT cells

**Figure 5:**
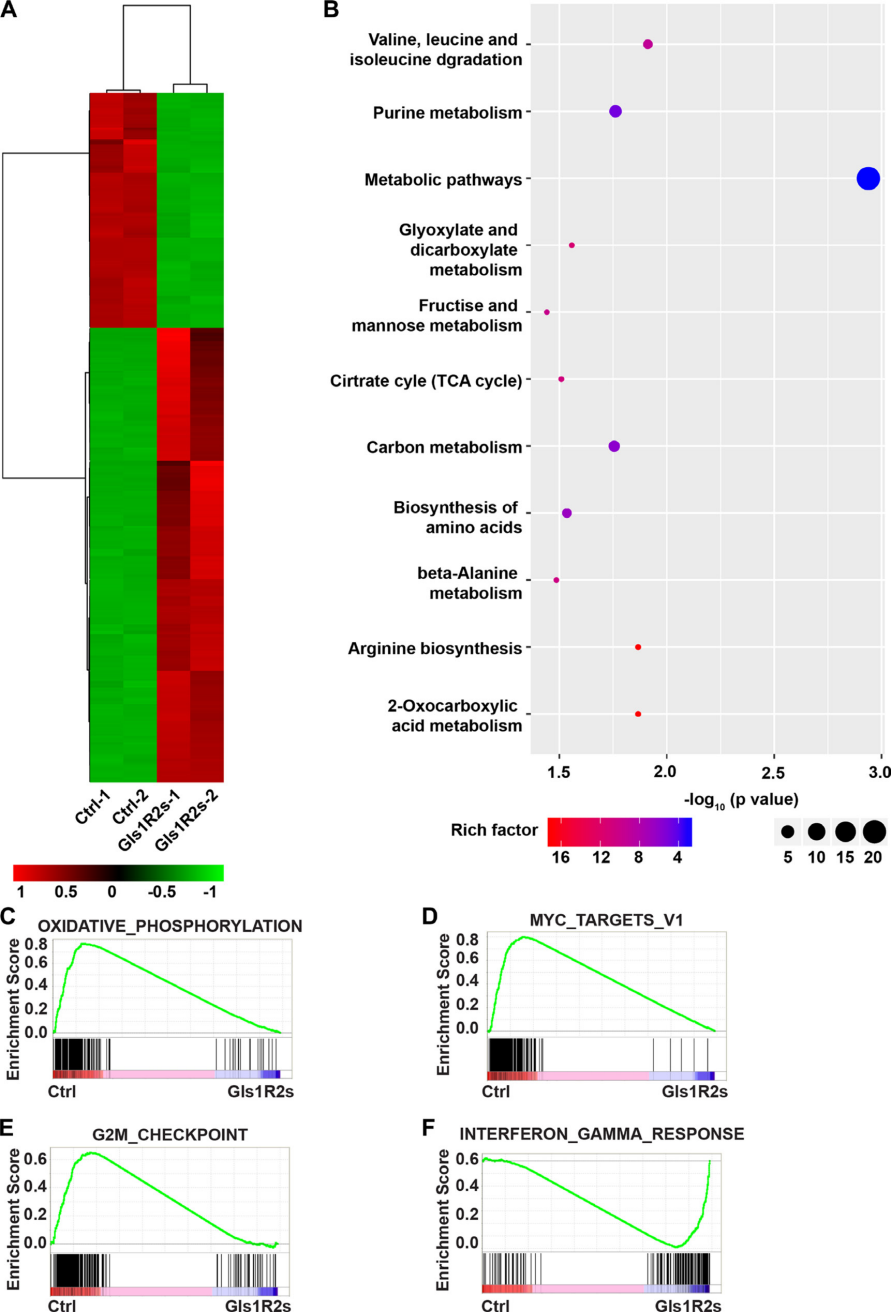
Gene expression signatures of MK-Gls1R2s-transduced PCT cells. (**A**) Heat map showing the expression changes of 414 genes and hierarchical clustering of the genes in MK-Gls1R2s-transduced PCT cells from two biological replicates. (**B**) KEGG pathway analysis of genes differentially expressed between MK-LAZR- and MK-Gls1R2s-transduced PCT cells. (**C**–**F**) Enriched gene sets with down- or up- regulation in MK-Gls1R2s-transduced PCT cells.

To further reveal the functions of Gls1 in PCT pathogenesis, transcriptome RNA-Seq was performed with PCT cells isolated from diseased recipients in MK-LAZR and MK-Gls1R2s groups. Genes were considered significantly altered, based on a 2-fold or greater change in mean expression (*p <* 0.01). Totally, 414 genes were determined to be significantly altered in expression (Figure 5A). In tumor cells, glutamine can be metabolized to enter the citric acid cycle to satisfy bioenergetic demands and macromolecular synthesis [27]. KEGG analysis showed that genes involved in metabolic pathways and biosynthesis were significantly enriched and down-regulated in Gls1 knockdown cells (Figure 5B). For example, TCA cycle, amino acids metabolism and biosynthesis, Carbohydrate metabolism were obviously restricted. Similar results were achieved by using GSEA analysis. For example, genes involved in “Oxidative phosphorylation” and “Glycolysis” were enriched as down-regulated (Figure 5C and Supplementary Table 1).

Whole transcriptome comparisons using GSEA analysis showed that MYC target genes were enriched as down-regulated in Gls1 knockdown cells (Figure 5D, Supplementary Figure 1B). MYC signaling pathway is essential for cancer stem cell self-renewal and cell survival. In addition, E2F targets were enriched and down-regulated (Supplementary Table 1), and mTORC1 signaling (Supplementary Figure 1C), an important pathway for cell survival, was also enriched as down-regulated. As an outcome of these impeded pathways, genes involved in the G2M checkpoint were enriched as down-regulated (Figure 5E). Genes involved in interferon or TNFα response were enriched as up-regulated (Figure 5F and Supplementary Figure 1D, 1E). As hall markers of antitumor activity, genes involved in viral infection and inflammation response, such as “viral myocarditis”, “HTLV-I infection”, “NF-kappa B signaling pathway”, and “Rheumatoid arthritis”, were significantly enriched as up-regulated (Supplementary Figure 1A and Supplementary Table 2).

### Combination of GLS1 inhibitor with other therapeutics achieved synergistic cytotoxic effects on MM cells

**Figure 6:**
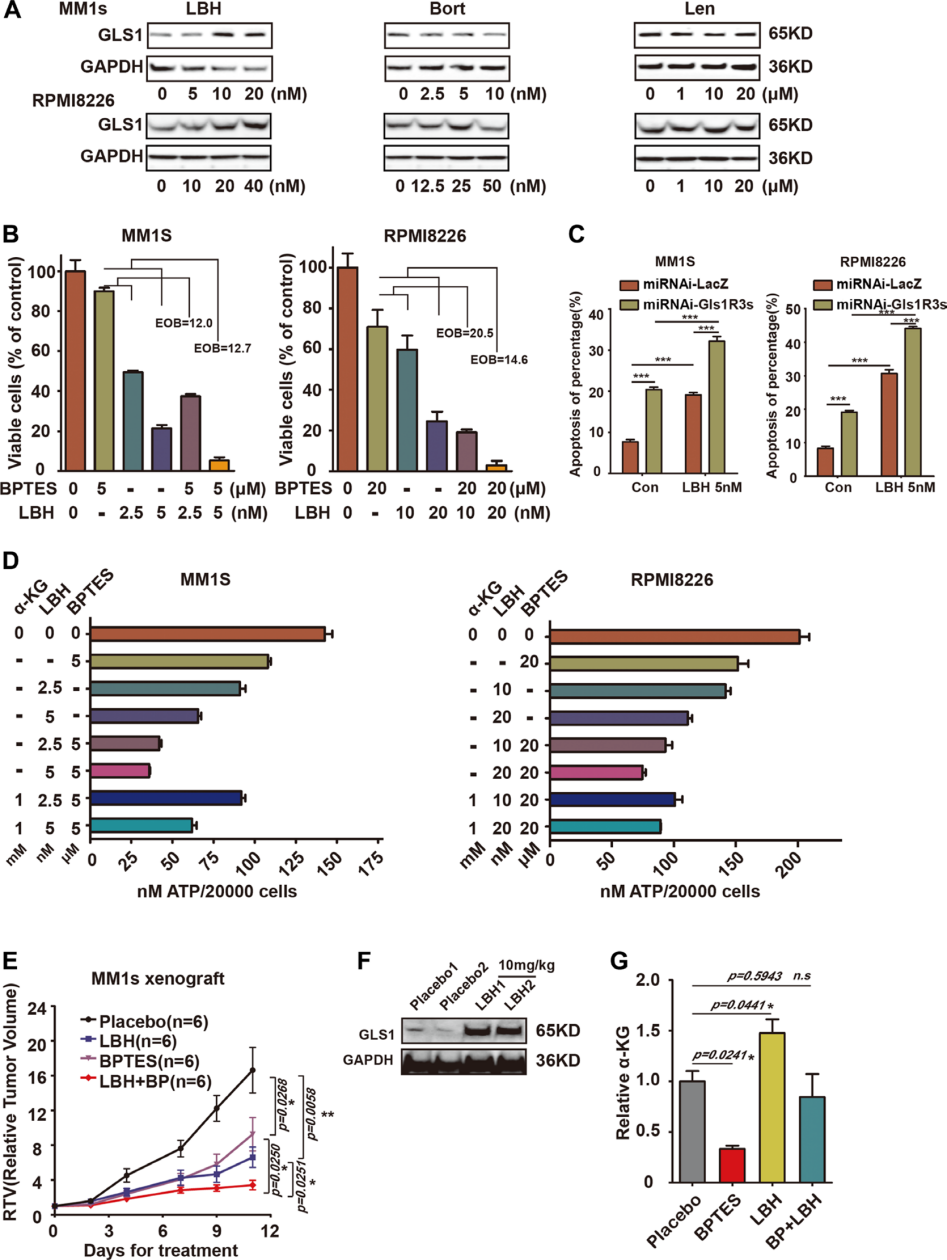
Combination of targeting GLS1 and current MM therapy drugs results in synergistic cytotoxic effects in MM cells. (**A**) Expression of GLS1 in MM1s and RPMI8226 cells exposed to LBH, Bort, and Len for 24 h at indicated concentrations. (**B**) The synergistic cytotoxic effect of BPTES and LBH on MM1s and RPMI8226 cells after 24h treatment, EOB>10 connotes synergy. (**C**) MM1S and RPMI8226 cells infection with miRNA-LacZ or miRNAi-Gls1R3s virus, then treated with 5 nM LBH, after 48 h, using APC-Annexin-V/PI kit analysis cell apoptosis. (**D**) Viability of cells treated with BPTES and LBH/or α-KG was measured with ATP activity. Each treatment was performed in triplicate in three independent experiments and presented as mean ± SE. (**E**) Xenografts were treated with vehicle (black line, *n* = 6), BPTES (10 mg/kg i. p., purple line, *n* = 6), LBH (10 mg/kg i. p., blue line, *n =* 6), or BPTES/LBH (i. p., red line, *n* = 6) for 10 days, and tumor volume was calculated. Data are represented as the relative change in tumor volume (RTV) (T0) ± SEM of 6 mice per group. The difference in RTV is highly significant as labeled. (**F**) Western-blot of GLS1 for cells isolated from vehicle or LBH589 treated tumors. Tumor numbers are indicated. GAPDH served as the western-blot loading control. (**G**) The concentration of α-KG in treated tumors (*n =* 3, in each groups) was measured with MS, and data were presented with the relative to that in Vehicle treated tumors. With *P* < 0.05^*^, *P <* 0.01^**^ and *P <* 0.001^***^ considered significant.

Based on the above results, we concluded that it was impossible to target Gls1 alone for MM cure. An alternative was to combine GLS1 inhibitor (s) with other drugs for potential MM treatment (s). According to previous results achieved in our laboratory, we found that the widely used therapeutics, such as LBH589 (Panobinostat), a potent and broad spectrum of HDACi, Bortezomib (Velcade), a proteasome inhibitor, and Lenalidomide (Revlimid), an immunomodulatory drug, increased GLS1 protein in tumor lines. We also found similar phenomes in MM lines (Figure 6A, 6F, and Supplementary Figure 3B). To investigate the syngeneic effects of combinations of GLS1 inhibitor (BPTES) with other therapeutics, MM1s and RPMI8226 cells were treated with BPTES combined with LBH, Bort, or Len, respectively. Consistent with previous reports, all compounds alone significantly impaired the survival of MM1s or RPMI8226 cells (Supplementary Figure 2A, 2B). Combinations of BPTES with any of these drugs achieved synergistic effects (Figure 6B, Supplementary Figure 2C, 2E and 2F). Moreover, Down-regulation of Gls1 with miRNAi combined with LBH or Bort also increased significantly more cell apoptosis compared with scramble (Figure 6C and Supplementary Figure 2D). To eliminate the possibility that the synergistic effects of BPTES came from off target effects, alpha-Ketoglutaric acid (α-KG), which is produced by deamination of glutamate and is used to represent the activity of GLS1, was added in the culture medium during treatment. α-KG can partially restore the cytotoxic effects triggered by the combination of BPTES and LBH (Figure 6D). These results indicated that the synergistic effects of BPTES with the therapeutics were contributed by specifically targeting GLS1.

In consideration of the therapeutic effects derived from combining BPTES with LBH in *in vivo*, we established xenografts with MM1s cells. Xenografts were treated with LBH, BPTES, or BPTES combined with LBH, respectively. Tumors grew more slowly in LBH- and BPTES-treated groups compared to vehicle-treated group, while the combination of BPTES and LBH achieved the greatest therapeutic effects (Figure 6E). To monitor the activity of BPTES, the concentration of α-KG was measured in *in vivo* tumors and α-KG was dramatically decreased in BPTES treated tumors, but was increased in LBH-treated tumors. BPTES could significantly reduce its level in LBH-treated tumors (Figure 6G). Similar results were achieved in Len and BPTES treated xenografts (Supplementary Figure 3A–3C).

## DISCUSSION

In this study, we showed that Gls1 played an important role for MM cell metabolism and survival and also was involved in PCT pathogenesis. We revealed that targeting Gls1 alone is not sufficient to prevent PCT develop or eliminate MM cells *in vivo;* however, a combination of BPTES with LBH, Bort, or Len can achieve synergic antitumor effects on MM both *in vitro* and *in vivo*.

Previous studies about GLS1’s biologic function were based on data from established tumor cells [6, 10, 14, 28–30]. Taking advantage of the PCT mouse model, we demonstrated that targeting Gls1 impeded pathogenesis of cMYC/KRAS12V-transduced PCT, but did not prevent mice from developing diseases (Figure 3A). Our results indicated that during cancer re-programming, inhibiting GLS1 activity or reducing glutamine intake could partially prevent cancer development. This conclusion was based on results from the effects of Gls1 mRNA knockdown or its inhibitor. It may be possible that completely inhibiting GLS1 activity can prevent cancer cell reprograming or get rid of cancer cells *in vivo*. However, considering the fact that mice lacking Gls1 died shortly after birth [31]. Treatments involving inhibition of GLS1 activity must be highly targeted to cancer cells to avoid side effects.

IRF4 is an essential transcription factor for plasma cell terminal differentiation [32], but its expression was not further increased in cMYC/KRAS12V-transformed BaF3 cells. One possible explanation is that IRF4 targets already were up-regulated in transformed cells, such as MYC and its targets, which was a primary target of IRF4 in myeloma cells [15]. The changed pattern of examined genes in cMYC/KRAS12V-transformed BaF3 cells with Gls1 knockdown is not the same as it is in PCT cells (Figure 2F and Figure 4B–4D). The difference may be partially due to cell types and growth conditions. The expression of Xbp1s was increased under all conditions (Figure 2D, F and Figure 4B). This may be because both of endoplasmic reticulum (ER) stress and plasma cell maturation contribute to the expression of *Xbp1*.

GLS1 protein level dramatically increased in LBH or Len monotherapy treated cells *in vivo,* than *in vitro* (Figure 6A, 6F, and Supplementary Figure 3B). We postulate that long-term ongoing stress increases the Warburg effect *in vivo*, suggesting that it is worthy to additionally target GLS1 when patients are treated with LBH or Len. If this phenomenon is found to be universal, targeting Warburg effect may broadly benefit cancer patients receiving chemotherapy.

Although our data did not support targeting GLS1 as a monotherapy for MM, GLS1 is still an attractive therapeutic target as part of a combinatorial treatment. From gene profiling analysis, we showed that the most important signal molecules which are critical for most cancer cell or MM survival, such as MYC, E2F, RAS, NFκB, IRF4, mTORC1, and others were significantly down-regulated when Gls1 level was reduced or its activity inhibited.

One striking finding was that a few recipients died of B cell leukemia, both in the MK group and MKGlsR1 group (Figure 3A). The recipients with B-ALL could have died because transduced donor cells contained some pro/pre-B cells which were transformed into leukemia cells by cMYC/KRAS12V. Interestingly, the progression of B-ALL in MKGlsR1 group was also slower. It appeared that Gls1 knockdown may also impede cMYC/KRAS12V-transduce B-ALL progression. Thus far, the biological effects of targeting Gls1 have not yet been evaluated in B-ALL.

During GSEA analysis, there was no any specific gene set associated with plasma cells or critical for MM survival that were significantly enriched, indicating that targeting Gls1 is not specific for MM. This is consistent with the observation that the Warburg effect is a hallmark of cancer cell metabolism in general and not restricted to a specific cell type [30].

## MATERIALS AND METHODS

### DNA constructs

The oligo sequences targeting human and mouse Gls1 mRNA (miGls1R1: 5′-GTCTGTTACCTAGCTTGGAAG-3′, miGls1R2 5′-AGATGGTGTCATGCTAGACAA-3′, and miGls1R3 5′-ATGGTGGTTTCTGCCCAATTA-3′) were cloned into the vector containing cMYC-2a-eGFP-IRES-KRAS12V, miGIs1Rs means miRNA targeting GIs1 sequence repeats. Knockdown efficiency was assessed following retroviral transduction into BaF3 cells as previously described [23].

### Cell lines and reagents

MM1s and RPMI8226 were cultured in RPMI1640 medium with 10% FBS (Gibco, ThermoFisher Scientific) and 100 units/ml penicillin/streptomycin (Gibco). Mouse cell line BaF3 was cultured in RPMI1640 medium (Gibco) with 10% FBS, 100 units/ml penicillin/streptomycin, 50 μM β-mercaptoethanol (Sigma-Aldrich), and 10% WEHI3 cell culture supernatant. GLS1 inhibitor BPTES (MB2348) and the chemotherapeutic reagents including LBH589 (LBH, MB5709), Bortezomib (Bort, MB1040), and Lenalidomide (Len, MB1136) were purchased from Meilubio (Dalian, China)

### Mouse models

All animal studies were performed in accordance with guidelines approved by the Institutional Animal Care and Use Committees of Sichuan University. PCT mouse model was made as previously described [16]. For the xenografts, 8–10-week-old female NOD/SCID mice were subcutaneously inoculated with 5 × 10^6^ MM1s cells. Treatment was initiated when mean tumor volumes reached 150 mm^3^. Mice were dosed via IP injection at 10 mg/kg of BPTES (10% DMSO in PBS) with LBH (dissolved in PBS), or 15 mg/kg of Len (dissolved in PBS) every other day and dosing was carried out for 12 days. Mice were euthanized when tumors reached 15 mm in diameter. Data were represented as the ratio of the final tumor volume relative to the initial tumor volume [(T/T0) × 100].

### Flow cytometry

Cells were obtained from peripheral blood (PB), spleen (SPL), bone marrow (BM), peritoneal tumor, and ascites from recipient mice and stained for the combination of the following antibodies: IgM-PE, B220-PECy7, CD138-APC, and CD38-PE (ThermoFisher Scientific). Samples were analyzed with FACS on a Fortesa machine (Becton Dickinson, NJ, U. S. A.) using Cell Quest software (Becton Dickinson). FACS data were analyzed and represented with Flowjo10.

### Histopathology and immunohistochemistry

Tissues were fixed, processed, sectioned, and stained with hematoxylin-eosin (H&E) by routine methods.

### Immunoblotting

Western blot was performed as a routine way. GLS1 antibody was purchased from Abcam (ab93434). P21(sc-6246) and P27(sc-1641) were purchased from Santa Cruz. Antibodies against MYC (RT1149), KRAS (ER40115), GFP (ET1604-26), β-actin (M1210-2), and GAPDH (EM1101) were purchased from Hangzhou Huaan Biotechnology (Hangzhou, China).

### Cell viability and apoptosis assays

BPTES, LBH, Bort, and Len were dissolved in DMSO and stored at –20° C. MM cells were seeded in a 96-well plate and compounds were added at indicated concentrations. Cell viability was measured with MTT at indicted time points with Biotek Cytation 3 at 570 nm. Synergy was computed using the Excess over Bliss (EOB) method. Bliss independence was determined using the formula C= A+B-A^*^B; where C designates the combined response for the 2 single compounds with effects A and B. An EOB>10 connotes synergy [33]. Apoptosis analysis using Annexin-V/PI detection kit APC purchased from Thermofisher (88800772).

### Gene expression profiling

PCT cells were isolated from diseased recipient ascites. RNA-seq was performed with Illumina MiSeq system. Gene set enrichment analysis (GSEA) was performed in Molecular Signatures Database (MSigDB, Broad Institute, Cambridge, MA, USA) [34, 35].

### Statistics

All experiments were performed in triplicate and repeated 2-3 times. Groups were compared using the *t*-test (GraphPad Prism software version 6.0), with *P* < 0.05^*^, *P <* 0.01^**^ and *P <* 0.001^***^ considered significant.

## SUPPLEMENTARY MATERIALS



## Abbreviations

MM: Multiple myeloma
GLS1: glautaminase1
PCT: plasmacytoma
α-KG: α-ketoglutarate
MGUS: monoclonal gammopathy of undetermined significance
BME: β-mercaptoethanol
LBH: LBH589
Bort: Bortezomib
Len: Lenalidomide
PB: peripheral blood
SPL: spleen
BM: bone marrow
EOB: Excess over Bliss
GSEA: Gene set enrichment analysis
KEGG: Kyoto Encyclopedia of Genes and Genomes
