# Supplementary material for: Targeting glutaminase1 and synergizing with clinical drugs achieved more promising antitumor activity on multiple myeloma
Source: Oncotarget. 2019 Oct 15;10(57):5993–6005. doi: 10.18632/oncotarget.27243 (PMC6800263; doi:10.18632/oncotarget.27243)
Supplement: Supplementary file 1 [file oncotarget-10-5993-s001.pdf]

Targeting glutaminase1 and synergizing with clinical drugs achieved more promising antitumor activity on multiple myeloma

SUPPLEMENTARY MATERIALS

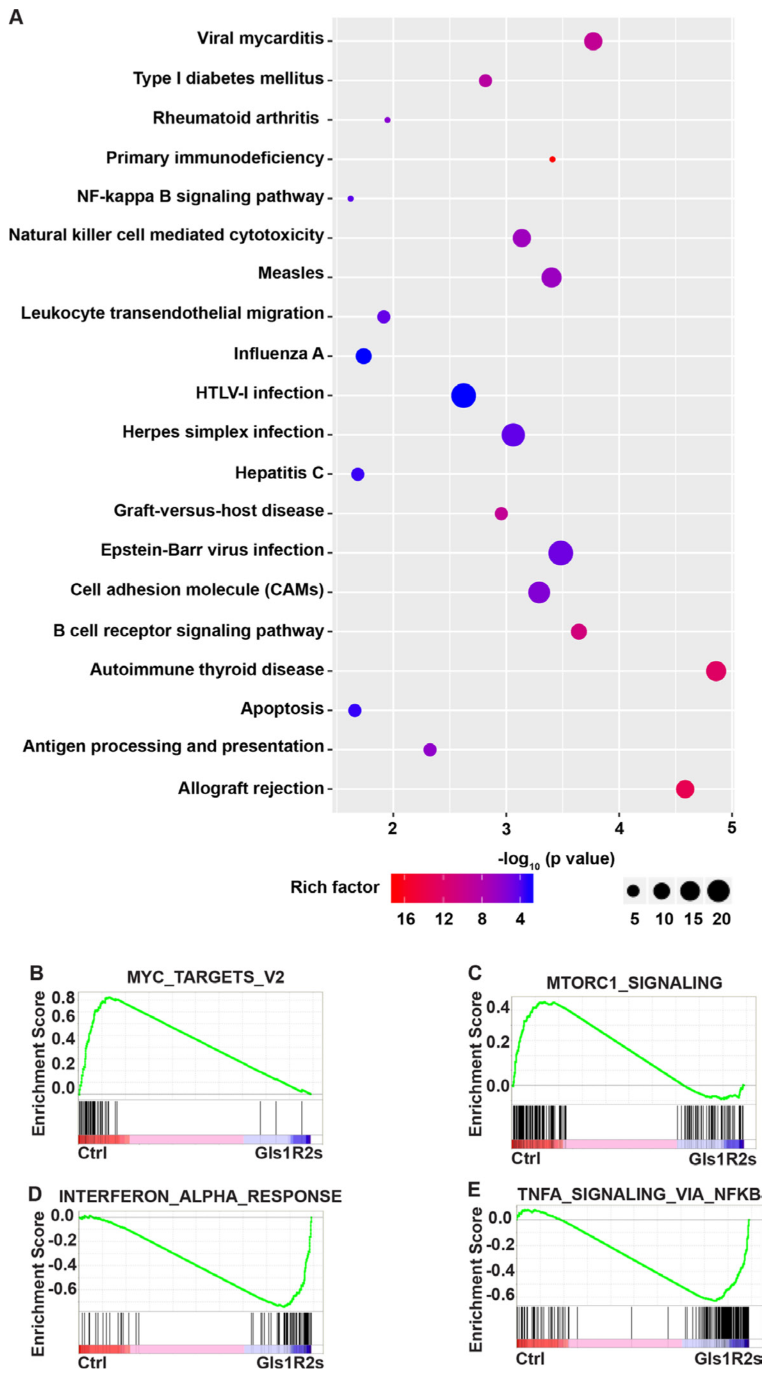

Supplementary Figure 1: Gene expression signatures of MK-Gls1R2s-transduced PCT cells. (**A**) KEGG pathway analysis of genes differentially expressed between MK-LAZR- and MK-Gls1R2s-transduced PCT cells. (**B–E**) Enriched gene sets with down- or up- regulation in MK-Gls1R2s-transduced PCT cells.

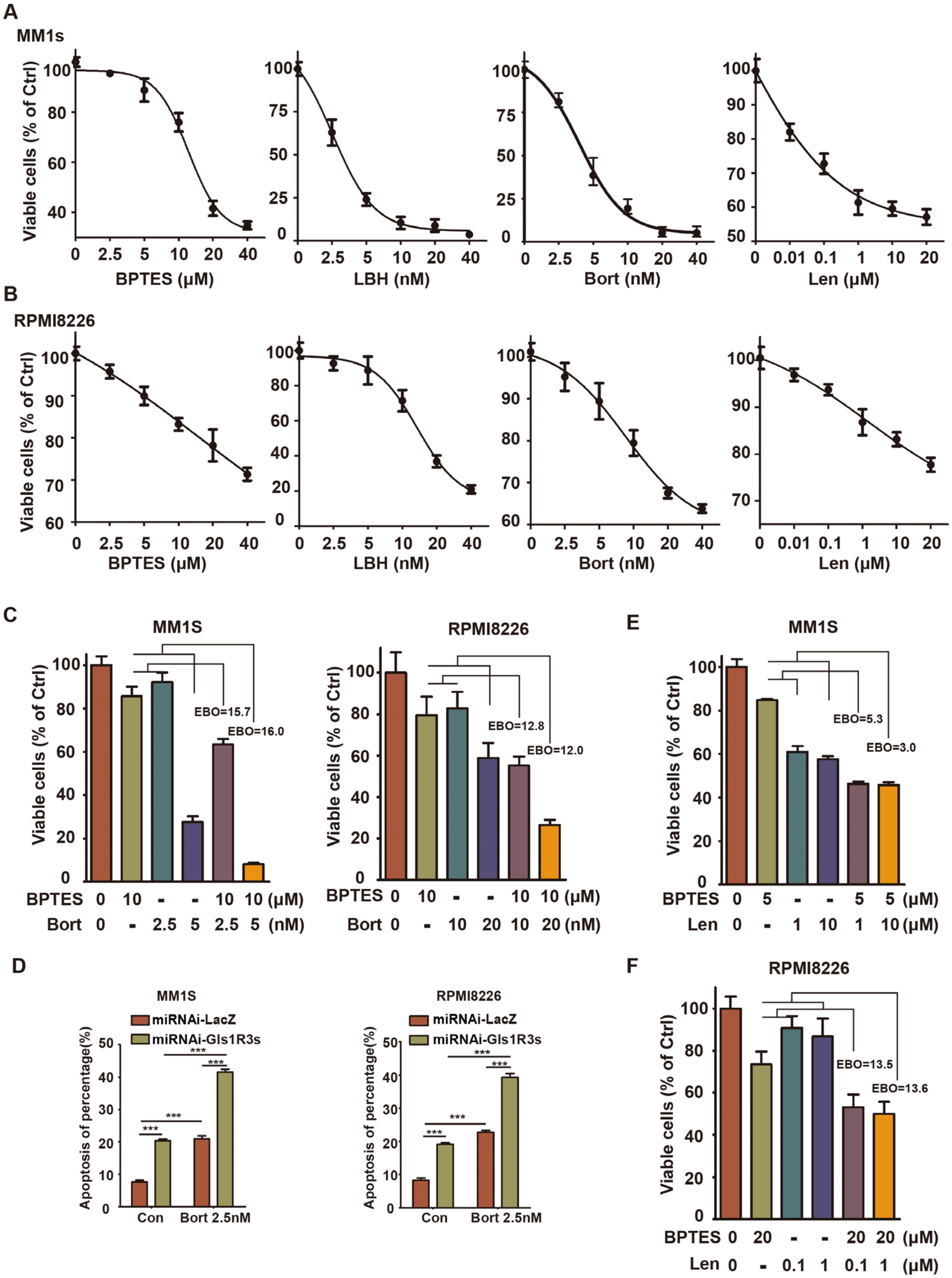

Supplementary Figure 2: The synergistic cytotoxic effect of the combination of targeting GLS1 and current MM therapy drugs on MM cells. (**A**) and (**B**) Dose responses of BPTES, LBH589, Bortezomib, and Lenalidomide on MM1s and RPMI8226 cells. Independent experiments were performed twice and presented as mean ± SE. (**C**) The synergistic cytotoxic effect of BPTES and Bortezomib on MM1s and RPMI8226 cells treated for 24 h. Cell viability was measured with MTT. Individual compound concentrations are indicated, EOB>10 connotes synergy. (**D**) MM1S and RPMI8226 cells infection with miRNA-LacZ or miRNAi-Gls1R3s virus, then treated with 2.5 nM Bort, after 48 h, using APC-Annexin-V/PI kit analysis cell apoptosis. (**E–F**) The synergistic cytotoxic effects of BPTES and Lenalidomide on MM1s and RPMI8226 cells for 24 h. Cell viability was measured with MTT. Individual compound concentrations are indicated and each treatment was performed in triplicate in three independent experiments and presented as mean ± SE, EOB>10 connotes synergy. With $P < 0.05^{*}$, $P < 0.01^{**}$ and $P < 0.001^{***}$ considered significant.

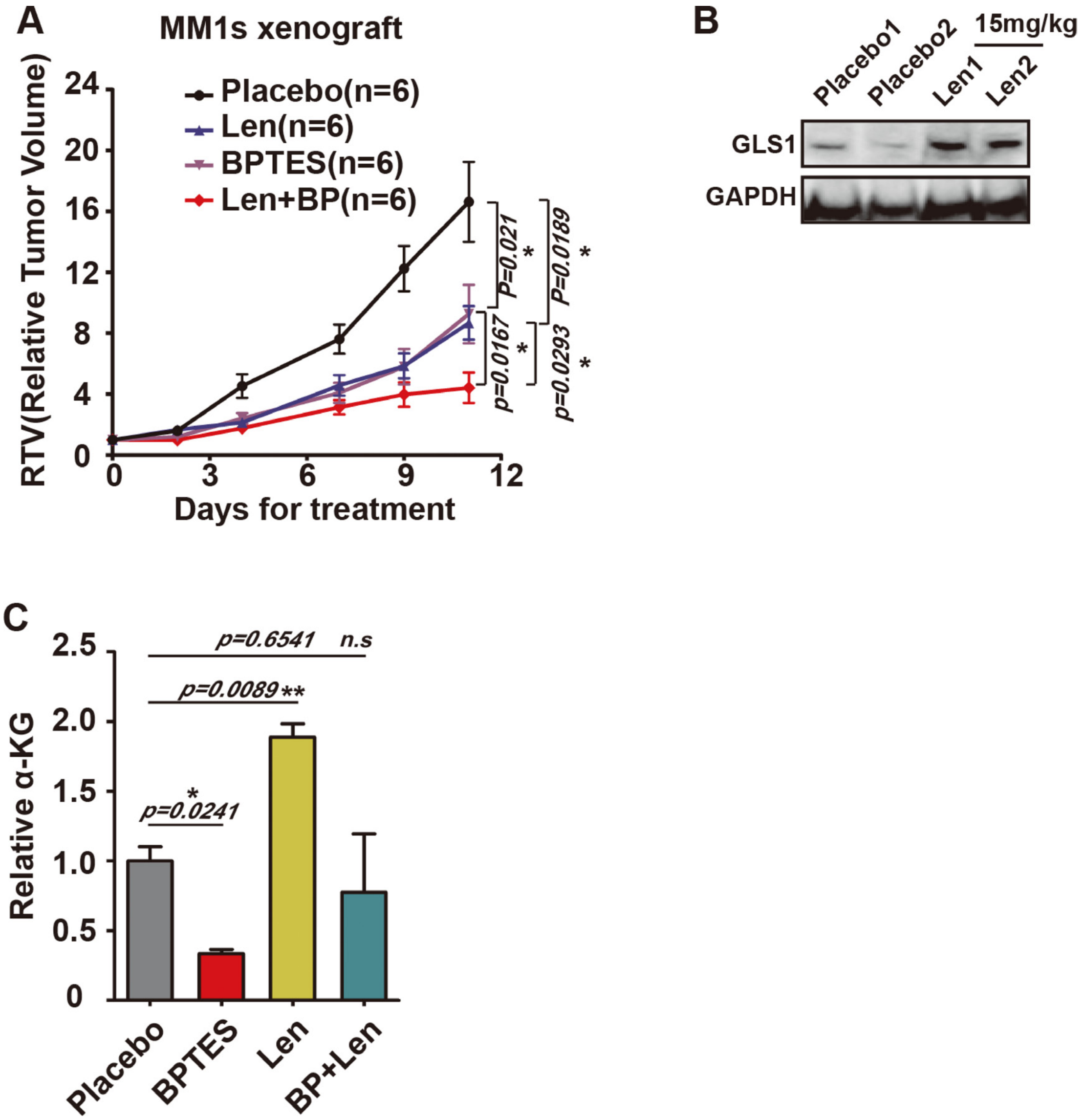

Supplementary Figure 3: The synergistic cytotoxic effect of the combination of a GLS1 inhibitor and current MM therapy drugs on MM cells. (**A**) Mice bearing MM1s xenografts were treated with vehicle (black line, n = 6), BPTES (10 mg/kg i. p., purple line, n = 6), Lenalidomide (15 mg/kg i. p., blue line, n = 6), or BPTES/ Lenalidomide (red line, n = 6) for 10 days, and tumor volume was calculated. Data are represented as the folds change in tumor volume (T0) ± SEM of 6 mice per group. The difference in RTV is highly significant as labeled. (**B**) Western-blot of GLS1 for cells isolated from vehicle or Lenalidomide treated tumors. Tumor numbers are indicated. GAPDH served as the western-blot loading control. (**C**) The concentration of α-KG in treated tumors (n = 3, in each groups) was measured with MS, and data were presented with the relative to that in vehicle treated tumors. Statistically significant differences are labeled as indicated.

Supplementary Table 1: Significantly downregulated gene set list from GSEA analysis of whole gene expression of MK-Gls1R2s-transduced PCT cells compared to MK-LAZR-transduced PCT cells in Molecular Signatures Database

Gene Set	SIZE	NES	NOM *p*-val	FDR *q*-val
MYC_TARGETS_V1	197	2.20	0	0
OXIDATIVE_PHOSPHORYLATION	192	2.11	0	0
MYC_TARGETS_V2	58	1.97	0	0
E2F_TARGETS	194	1.95	0	0
G2M_CHECKPOINT	194	1.78	0	0.00
ADIPOGENESIS	194	1.65	0	0.00
FATTY_ACID_METABOLISM	147	1.59	0	0.00
MTORC1_SIGNALING	193	1.55	0	0.01
PEROXISOME	99	1.43	0	0.02
SPERMATOGENESIS	131	1.31	0.04	0.07
REACTIVE_OXIGEN_SPECIES_PATHWAY	43	1.27	0.11	0.10
DNA_REPAIR	138	1.24	0.07	0.12
MITOTIC_SPINDLE	196	1.18	0.09	0.19
GLYCOLYSIS	193	1.13	0.15	0.26
ANDROGEN_RESPONSE	94	1.10	0.25	0.32
XENOBIOTIC_METABOLISM	185	1.09	0.21	0.31
PANCREAS_BETA_CELLS	39	0.94	0.54	0.74
PI3K_AKT_MTOR_SIGNALING	103	0.93	0.61	0.73
UNFOLDED_PROTEIN_RESPONSE	112	0.92	0.66	0.74
BILE_ACID_METABOLISM	110	0.90	0.69	0.75
NOTCH_SIGNALING	31	0.83	0.75	0.91

Significantly downregulated gene set list from GSEA analysis of whole gene expression of MK-Gls1R2s-transduced PCT cells compared to MK-LAZR-transduced PCT cells in Molecular Signatures Database.

Supplementary Table 2: Significantly upregulated changed gene set list from GSEA analysis of whole gene expression of MK-Gls1R2s-transduced PCT cells compared to MK-LAZR-transduced PCT cells in Molecular Signatures Database

Gene set	SIZE	NES	NOM *p*-val	FDR *q*-val
INTERFERON_GAMMA_RESPONSE	184	−1.95	0	0
INTERFERON_ALPHA_RESPONSE	88	−1.90	0	0
TNFA_SIGNALING_VIA_NFKB	195	−1.78	0	0.00
EPITHELIAL_MESENCHYMAL_ TRANSITION	192	−1.69	0	0.00
ALLOGRAFT_REJECTION	183	−1.56	0	0.01
INFLAMMATORY_RESPONSE	194	−1.50	0	0.02
IL6_JAK_STAT3_SIGNALING	85	−1.44	0.01	0.03
APICAL_JUNCTION	192	−1.35	0.01	0.08
APOPTOSIS	156	−1.33	0.02	0.09
COMPLEMENT	180	−1.32	0.01	0.08
IL2_STAT5_SIGNALING	191	−1.30	0.02	0.09
COAGULATION	131	−1.28	0.04	0.11
HYPOXIA	192	−1.27	0.02	0.10
KRAS_SIGNALING_UP	190	−1.27	0.02	0.10
KRAS_SIGNALING_DN	187	−1.26	0.04	0.10
MYOGENESIS	197	−1.23	0.04	0.12
ESTROGEN_RESPONSE_LATE	191	−1.14	0.13	0.27
ESTROGEN_RESPONSE_EARLY	196	−1.09	0.22	0.38
WNT_BETA_CATENIN_SIGNALING	41	−1.07	0.33	0.41
HEDGEHOG_SIGNALING	35	−1.05	0.35	0.45
HEME_METABOLISM	181	−1.04	0.29	0.45
UV_RESPONSE_DN	141	−1.04	0.32	0.45
PROTEIN_SECRETION	94	−1.01	0.41	0.51
P53_PATHWAY	193	−1.00	0.41	0.52
TGF_BETA_SIGNALING	54	−0.94	0.55	0.70
UV_RESPONSE_UP	149	−0.94	0.63	0.70
APICAL_SURFACE	44	−0.89	0.68	0.86
ANGIOGENESIS	35	−0.83	0.77	0.97
CHOLESTEROL_HOMEOSTASIS	69	−0.82	0.86	0.95

Significantly upregulated changed gene set list from GSEA analysis of whole gene expression of MK-Gls1R2s-transduced PCT cells compared to MK-LAZR-transduced PCT cells in Molecular Signatures Database.
